# Neurovisceral phenotypes in the expression of psychiatric symptoms

**DOI:** 10.3389/fnins.2015.00004

**Published:** 2015-02-10

**Authors:** Jessica A. Eccles, Andrew P. Owens, Christopher J. Mathias, Satoshi Umeda, Hugo D. Critchley

**Affiliations:** ^1^Psychiatry, Brighton and Sussex Medical SchoolBrighton, UK; ^2^Sussex Partnership National Health Service Foundation TrustBrighton, UK; ^3^National Hospital Neurology and Neurosurgery, UCL National Health Service TrustLondon, UK; ^4^Institute of Neurology, University College LondonLondon, UK; ^5^Department of Psychology, Keio UniversityTokyo, Japan; ^6^Sackler Centre for Consciousness Science, University of SussexFalmer, UK

**Keywords:** postural tachycardia syndrome, joint hypermobility, vasovagal syncope, anxiety, psychiatry

## Abstract

This review explores the proposal that vulnerability to psychological symptoms, particularly anxiety, originates in constitutional differences in the control of bodily state, exemplified by a set of conditions that include Joint Hypermobility, Postural Tachycardia Syndrome and Vasovagal Syncope. Research is revealing how brain-body mechanisms underlie individual differences in psychophysiological reactivity that can be important for predicting, stratifying and treating individuals with anxiety disorders and related conditions. One common constitutional difference is Joint Hypermobility, in which there is an increased range of joint movement as a result of a variant of collagen. Joint hypermobility is over-represented in people with anxiety, mood and neurodevelopmental disorders. It is also linked to stress-sensitive medical conditions such as irritable bowel syndrome, chronic fatigue syndrome and fibromyalgia. Structural differences in “emotional” brain regions are reported in hypermobile individuals, and many people with joint hypermobility manifest autonomic abnormalities, typically Postural Tachycardia Syndrome. Enhanced heart rate reactivity during postural change and as recently recognized factors causing vasodilatation (as noted post-prandially, post-exertion and with heat) is characteristic of Postural Tachycardia Syndrome, and there is a phenomenological overlap with anxiety disorders, which may be partially accounted for by exaggerated neural reactivity within ventromedial prefrontal cortex. People who experience Vasovagal Syncope, a heritable tendency to fainting induced by emotional challenges (and needle/blood phobia), are also more vulnerable to anxiety disorders. Neuroimaging implicates brainstem differences in vulnerability to faints, yet the structural integrity of the caudate nucleus appears important for the control of fainting frequency in relation to parasympathetic tone and anxiety. Together there is clinical and neuroanatomical evidence to show that common constitutional differences affecting autonomic responsivity are linked to psychiatric symptoms, notably anxiety.

## Introduction

Influential theories argue that bodily states of arousal are a key component to emotions, and are the basis to emotional feeling states. Emotional processes are intrinsically coupled to autonomic bodily responses through shared neural substrates. Exaggerated patterns of autonomic responsivity can enhance the expression of panic or anxiety symptoms. Some of the vulnerability to psychological symptoms, particularly anxiety, originates in constitutional differences in the control of bodily states of arousal. Joint Hypermobility, Postural Tachycardia Syndrome and Vasovagal Syncope represent common conditions in which there is evidently a stronger association. The mechanisms underlying these brain-body interactions can be defined by combining brain imaging with detailed physiological monitoring of psychiatric and neurological patients and healthy controls. Ultimately, these interactions are relevant to the recognition, understanding and treatment of individuals with anxiety, and also for stress-sensitive medical disorders. By characterizing the interplay between brain and body in detail, clinically relevant insights can be gained into the mechanisms that underpin both adaptive and maladaptive psychological and physical states. This in turn will lead to targeted interventions for the personalized treatment of symptoms, for enhancing health and resilience and for mitigating adverse events, e.g., drug side effects. We argue that there are specific yet common constitutional variants (i.e., phenotypes) in physiological reactivity (i.e., related to the autonomic nervous system) that have major influences on emotional state and by extension on vulnerability to psychiatric disorder. Below we discuss the relevance of three of these “neurovisceral phenotypes” (Joint Hypermobility, Postural Tachycardia Syndrome and Vasovagal Syncope) to psychiatric symptoms.

## Neurovisceral phenotypes

### Joint hypermobility

#### Clinical picture

Joint hypermobility affects up to 20% of the general population (Mulvey et al., 2013) yet is often poorly recognized (Grahame, 2008). It is characterized by a variation in the type and distribution pattern of collagen. Autosomal dominant heritance is often observed, yet few genes are reliably implicated, and a preponderance amongst females suggests incomplete penetrance, influenced by sex (see Castori, 2012). Joint hypermobility reflects a disordered collagen ratio (notably Type I/III) (Child, 1986) and Joint Hypermobility *Syndrome* (synonymous with Ehlers-Danlos Hypermobility Type–formerly Type III) (see, Tinkle et al., 2009) describes the presence of joint hypermobility with other musculoskeletal and extra articular connective tissue difficulties (Grahame et al., 2000). See Table 1 for diagnostic criteria.

**Table 1:** **Diagnostic criteria of JHS/EDS III**.

**MAJOR CRITERIA**
Beighton score of 4/9 or greater (either currently or historically)
Arthralgia for longer than 3 months in 4 or more joints
**MINOR CRITERIA**
Beighton score of 1, 2 or 3/9 (0, 1, 2 or 3 if aged 50+)
Arthralgia (>3 months) in one to three joints or back pain (>3 months), spondylosis, spondylolysis/spondylolisthesis
Dislocation/Subluxation in more than one joint, or in one joint on more than one occasion
Soft tissue rheumatism. > 3 lesions (e.g., epicondylitis, tenosynovitis, bursitis)
Marfanoid habitus (tall, slim, span/Height ratio >1.03, upper: lower segment ratio less than 0.89, arachnodactyly [positive Steinberg/Wrist signs]
Abnormal skin: striae, hyperextensibility, thin skin, papyraceous scarring
Eye signs: drooping eyelids or myopia or antimongoloid slant
Varicose veins or hernia or uterine/rectal prolapse

*Joint Hypermobility Syndrome is diagnosed in the presence two major criteria, or one major and two minor criteria, or four minor criteria. Two minor criteria will suffice where there is an unequivocally affected first-degree relative*.

*Joint Hypermobility Syndrome is excluded by presence of Marfan or Ehlers-Danlos syndromes other than the hypermobility type of Ehlers-Danlos syndrome) as defined by the Ghent 1996 and Villefranche 1998 criteria respectively. Criteria Major 1 and Minor 1 are mutually exclusive as are Major 2 and Minor 2*.

Over recent years there has been growing recognition of extra-articular features of Joint Hypermobility: these affect almost every system of the body (unsurprisingly as collagen is not confined to joints). Conditions associated with joint hypermobility go beyond rheumatology to include Chronic Fatigue Syndrome (Nijs et al., 2006), Fibromyalgia (Ofluoglu et al., 2006), and Irritable Bowel Syndrome (Zarate et al., 2010). See Table 2 for extra-articular disorders associated with joint hypermobility.

**Table 2:** **Summarizes extra-articular disorders associated with joint hypermobility with example references**.

**Condition**	**References**
Attention deficit hyperactivity disorder	Koldas Dogan et al., 2011
Anxiety	See later review, e.g., (Martin-Santos et al., 1998)
Asthma	Morgan et al., 2007
Carpal tunnel syndrome	Aktas et al., 2008
Chiari malformation type I	Milhorat et al., 2007
Chronic constipation	De Kort et al., 2003
Chronic fatigue syndrome	Nijs et al., 2006
Chronic regional pain syndrome	Stoler and Oaklander, 2006
Crohn's disease	Vounotrypidis et al., 2009
Developmental co-ordination disorder	Kirby and Davies, 2007
Fecal incontinence	Arunkalaivanan et al., 2009
Fibromyalgia	Ofluoglu et al., 2006
Functional gastrointestinal disorder	Zarate et al., 2010
Headache attributed to spontaneous cerebrospinal fluid leakage	Schievink et al., 2004
Hiatus hernia	Al-Rawi et al., 2004
Mitral valve prolapse (MVP)	Yazici et al., 2004
Migraine	Bendik et al., 2011
New daily persistent headache	Rozen et al., 2006
Pelvic organ prolapse	Lammers et al., 2012
Postural tachycardia syndrome	Mathias et al., 2012
Psychological distress	Baeza-Velasco et al., 2011
Rectal evacuatory dysfunction	Mohammed et al., 2010
Somatosensory amplification	Baeza-Velasco et al., 2011
Urinary stress incontinence	Karan et al., 2004

*Extra-articular disorders associated with joint hypermobility, adapted from Castori (2012)*.

#### Association with psychiatric disorder

Psychiatric phenomena are increasingly recognized as another important extra-articular manifestation of joint hypermobility.

Bulbena et al. reported the first association between joint hypermobility and anxiety disorder in rheumatology patients in a case-control study (Bulbena et al., 1993), the overrepresentation of hypermobility in anxiety disorder patients was subsequently confirmed in later studies (e.g., Bulbena et al., 1996; Martin-Santos et al., 1998). Further studies have consistently demonstrated this association in non-clinical populations (e.g., Bulbena et al., 2004a,b) and in a large general population cohort (Bulbena et al., 2011). This association with anxiety is consistently replicated over the years (Bulbena et al., 1993; Garcia Campayo et al., 2010; Bulbena et al., 2011; Sanches et al., 2012).

A recent meta-analysis of 3597 subjects demonstrates consistently that in both healthy and clinical population's hypermobile people experience significantly more intense fears, agoraphobia, panic, anxiety, and depressive disorders (Smith et al., 2014).

Additionally significantly higher rates of joint hypermobility can be observed among patients with Bipolar Disorder and Neurodevelopmental disorders, such as Attention Deficit Hyperactivity Disorder and Autism Spectrum Disorder (Eccles et al., 2014a,b).

The underlying neurobiological mechanisms associating joint hypermobility with psychiatric disorders have not been studied in detail. However, brain and body are intrinsically and dynamically coupled; perceptions, emotions and cognitions respond to, and change, the state of the body. These interactions form much of the content of psychophysiological research identifying bodily signatures of mental activity. The central interaction of processes supporting the generation and the representation of autonomically-mediated changes in visceral state may be the critical mediator of autonomic influences on cognition and emotion. Central viscerosensory and visceromotor representations are exchanged as afference and efference copies to allow error signaling. Where there is mismatch between intended and actual autonomic state, corrective efferent reactions are accompanied by interpretative processes. The unconscious operation of autonomic nervous system can be interrupted by deviations from expected state, i.e., we become aware of our autonomic bodily state when we experience changes in internal state that are “unpredicted” by control centers (Seth et al., 2011; Critchley et al., 2013). In disorders that typically involve impaired collagen (present in blood vessels) such as joint hypermobility and postural tachycardia syndrome it is likely that reduced venous return during standing due to venous pooling or denervation causing low plasma volume may be responsible for a an increased sympathetic state–as the body attempts to compensate for these abnormalities—resulting in orthostatic intolerance and associated symptoms (Bohora, 2010; Benarroch, 2012; Mathias et al., 2012).

Thus, we postulate that a major factor is the dysregulation of the autonomic nervous system, which particularly drives the expression of anxiety, a pervasive symptom across almost all psychological disorders.

#### Evidence of autonomic dysfunction in Joint Hypermobility

Significant dysautonomia is observed in patients with Joint Hypermobility Syndrome. Symptoms related to the autonomic nervous system, such as syncope and presyncope, orthostatic intolerance, palpitations, chest discomfort, fatigue, and heat intolerance, are significantly more common among hypermobile patients. Orthostatic hypotension, postural tachycardia syndrome, and uncategorized orthostatic intolerance were found in over three-quarters of hypermobile patients compared to one tenth of controls. Patients with Joint Hypermobility Syndrome have a greater mean drop in systolic blood pressure during hyperventilation, and a greater increase in systolic blood pressure after a cold pressor test, than controls. They also have evidence of heighted vasoconstriction mediated by alpha-adrenergic and beta-adrenergic hyper-responsiveness (as assessed by administration of phenylephrine) as assessed by administration of isoproterenol) (Gazit et al., 2003).

Symptoms suggestive of autonomic dysfunction are also more common in patient with Joint hypermobility Syndrome. These include including presyncope, palpitations and gastrointestinal disturbances (Hakim and Grahame, 2004). Measures of heart rate reactivity and reactions to the Valsalva maneuver, during autonomic functional testing also indicate autonomic dysregulation in Joint Hypermobility Syndrome patients compared to controls, including (De Wandele et al., 2014).

**Figure 1:**
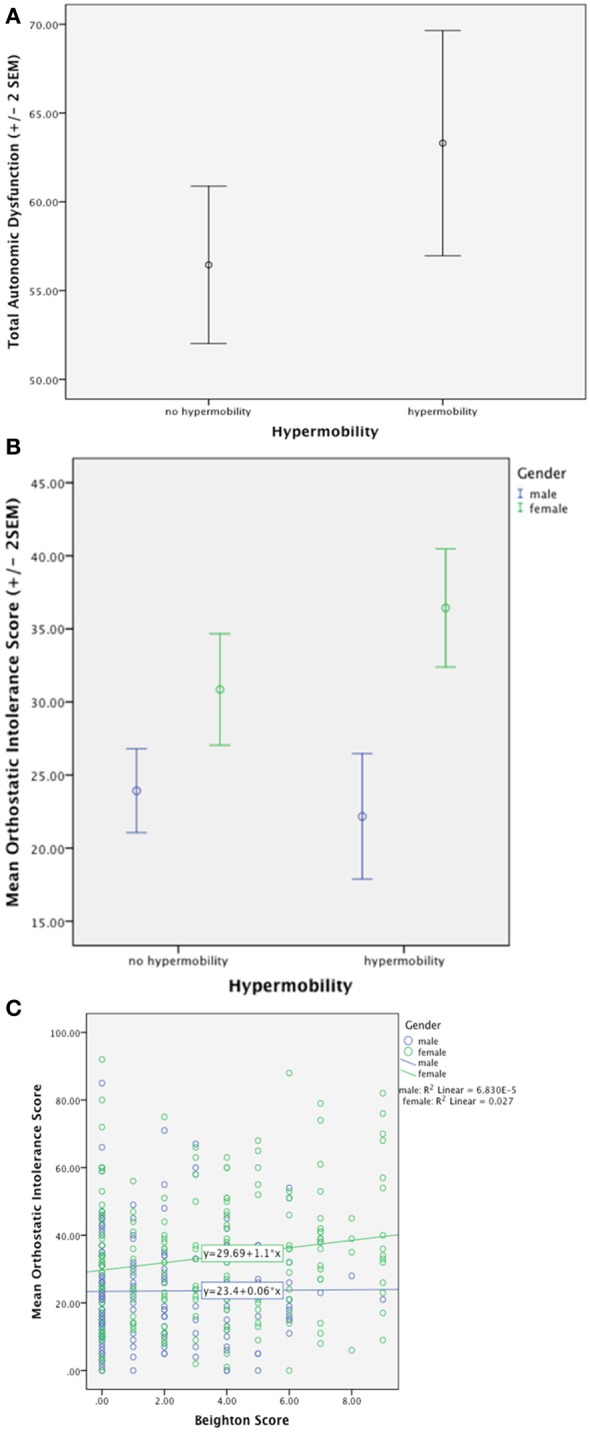
**(A)** Demonstrates significantly higher total autonomic dysfunction in hypermobile patients compared to non-hypermobile patients. **(B)** Shows significantly higher orthostatic intolerance in hypermobile patients compared to non-hypermobile patients with an effect of gender. **(C)** Demonstrates positive correlation with orthostatic intolerance and degree of hypermobility with an effect of gender (Eccles et al., 2014b).

A recent survey of 361 general psychiatric patients (Eccles et al., 2014b) demonstrates that symptoms of autonomic dysfunction are not only higher in hypermobile patients, but also correlate strongly with degree of hypermobility. See Figure 1. This association is driven further by an effect of gender, such that there are significant effects in women only. Postural Tachycardia Syndrome patients report increased dizziness throughout the menstrual cycle, particularly during menses, compared to healthy controls (Peggs et al., 2012). Estrogen affects blood volume (Pritchard, 1965) as well as the renin-angiotensin-aldosterone system and estrogen therapy has therefore been used to improve baroreflex regulation in post-menopausal women (Hunt et al., 2001). Orthostatic intolerance has been found to improve during the midluteal phase of the menstrual cycle in Postural Tachycardia Syndrome patients (Fu et al., 2010), this may be due to higher midluteal estrogen and progesterone levels upregulating renal-adrenal hormones (Stachenfeld and Taylor, 2005). This may go some way in explaining the gender differences in orthostatic symptoms associated with hypermobility. Overall, hypermobile patients had higher mean autonomic dysfunction and orthostatic intolerance than non-hypermobile patients. Formal mediation analysis suggests that orthostatic intolerance partially mediates the relationship between joint hypermobility and anxiety; again, an effect only seen in females.

Postural Tachycardia Syndrome is characterized by a marked rise in heart rate of 30 beats-per-minute or greater occurring within 10 min of head-up tilt or standing from supine, or a heart rate while upright of >120 beats per minute (Mathias et al., 2012). Postural Tachycardia Syndrome, in the UK at least, is very frequently associated with Joint Hypermobility Syndrome: ninety-six per cent of Postural Tachycardia Syndrome patients attending a UK tertiary autonomic disorders service have Joint Hypermobility (Owens et al., in preparation). There is a strong phenomenological overlap with anxiety disorders: Patients report symptoms similar to panic, including dizziness, palpitations and gastrointestinal disturbance. In an examination of 114 individuals with joint hypermobility syndrome, over 40% fulfilled criteria for Postural Tachycardia Syndrome (Hakim et al., 2009). Interestingly, healthy individuals with hypermobility report increased subjective sensitivity to autonomic bodily changes, a psychological trait that has been associated with an increased predisposition to anxiety disorder (Eccles et al., 2012).

#### Evidence of brain-body interactions from neuroimaging

**Figure 2:**
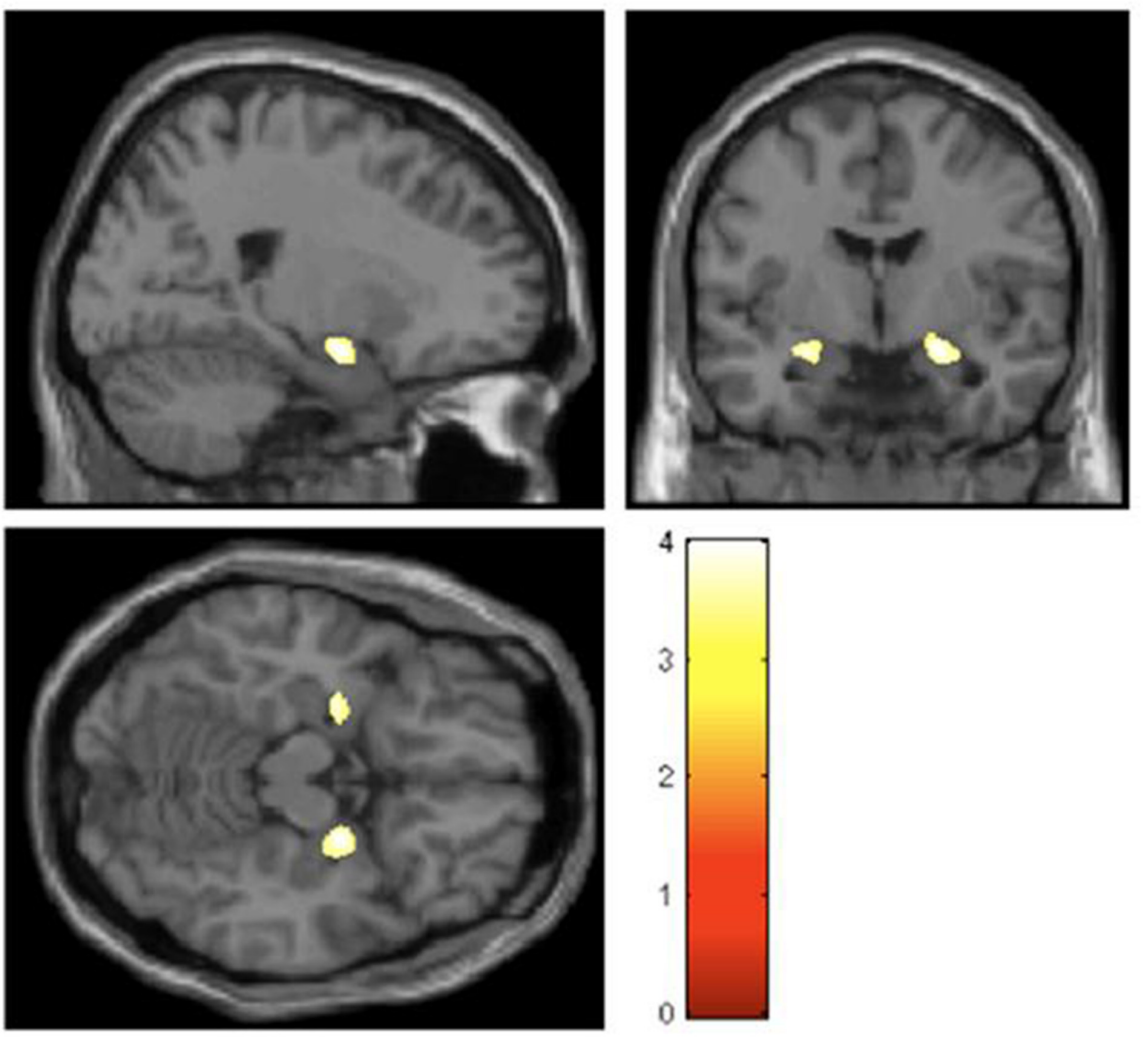
**Regions of gray-matter volume difference in hypermobile participants compared with the non-hypermobile group (threshold *P* < 0.001 uncorrected) (Eccles et al., 2012)**.

Structural differences in key emotion processing brain regions, notably affecting the amygdala bilaterally, are observed in these otherwise healthy individuals. The hypermobile group as a whole also display decreased anterior cingulate and left parietal cortical volume while the degree of hypermobility correlates negatively with both superior temporal and inferior parietal volume (Eccles et al., 2012). Figure 2 demonstrates bilateral differences in amygdala volume.

In a recent functional neuroimaging study of healthy volunteers, hypermobile, compared to non-hypermobile, participants displayed heightened neural reactivity to sad and angry scenes within brain regions implicated in anxious feeling states, notably insular cortex (Mallorqui-Bague et al., 2014).

#### Conclusions

These data suggest that specific brain regions mediate the interaction between psychological processes and the physiological state of the body, in a manner ultimately crucial to the generation of anxiety and related symptoms in the joint hypermobility phenotype. The amygdala is a key region supporting motivational and behaviors and emotional memory; it is implicated in threat processing, generation of bodily arousal reactions and the expression of mood symptoms. These neuroimaging data implicate the amygdala and insular cortices as the most likely neural substrate underlying the association between of hypermobility, clinical anxiety and psychosomatic disorders. Speculatively, potential mechanisms that may further account for the mediating role of amygdala between anxiety and hypermobility include heightened susceptibility to the threat of pain and a perturbation of autonomic afferent feedback (Nicotra et al., 2006). Hypermobility syndrome is associated with pain syndromes such as fibromyalgia, irritable bowel syndrome and chronic regional pain syndrome. Differences in amygdala reactivity are also reported for these (Tracey and Bushnell, 2009). Moreover, there is evidence to suggest that the autonomic nervous system is dysregulated in hypermobile individuals. Theoretically, anxiety is linked to the unpredictable states of bodily arousal, engaging regions such as amygdala and insula (Paulus and Stein, 2006). Observed differences in dorsal right anterior cingulate cortex were also observed. This region is implicated in autonomic reactivity, and engaged in the cognitive control of pain and negative feeling states (Critchley, 2009; Tracey and Bushnell, 2009).

Hypermobility was also linked to reduced right superior temporal volume. Right superior temporal cortex supports the sensory processing of social emotional information and structural abnormalities of this region are observed in autism (Boddaert et al., 2004), where neurodevelopmental emotional deficits are frequently accompanied by heightened anxiety. Hypermobility has been linked to autism (Eccles et al., 2014a).

Patients with joint hypermobility show heightened interoceptive sensibility compared to those without (Eccles et al., 2012) and interoceptive sensitivity has been show to mediate the relationship between state anxiety and hypermobility and it could be hypothesized combining functional neuroimaging with autonomic monitoring will show that inefficient neural co-ordination of efferent autonomic drive with imprecise interoceptive representations may be amplified in hypermobile individuals.

### Postural Tachycardia Syndrome

#### Clinical picture and autonomic dysfunction

Postural Tachycardia Syndrome is now recognized as one of the most common forms of orthostatic intolerance. As noted, it is characterized by an excessive heart rate increase of +30 beats per minute (beats per minute) or a heart rate of >120 beats per minute without orthostatic hypotension within the initial 10 min of orthostasis or head up tilt. Postural Tachycardia Syndrome has only relatively recently (1993) been clearly described (Schondorf and Low, 1993) yet reports of soldiers suffering with “irritable heart syndrome” and “soldier”s heart'—conditions that appear very similar to Postural Tachycardia Syndrome—date back to the American Civil War (Wooley, 1976). More recently, it is recognized that symptoms can also be provoked by food digestion, heat or moderate exercise in some Postural Tachycardia Syndrome patients (Mathias et al., 2012).

Postural Tachycardia Syndrome is a heterogeneous disorder: It can be broadly divided into hyperadrenergic or neuropathic phenotypes (see Table 3 Overview of Postural Tachycardia Syndrome phenotypes, adapted from Benarroch (2012). However, hypovolemia, abnormalities of the noradrenaline transporter (Esler et al., 2006; Lambert et al., 2008; Bayles et al., 2012), infection (Schondorf and Low, 1993), deconditioning (Parsaik et al., 2012), and poor orthostatic cerebral autoregulation (Ocon et al., 2009) are also implicated in Postural Tachycardia Syndrome pathophysiology. Postural Tachycardia Syndrome patients frequently present with other concurrent or comorbid autonomic symptoms. These include pre-syncope, syncope, dizziness, palpations, headache, fatigue, bladder, and gastrointestinal (GI) symptoms. As previously noted, around 90% of Postural Tachycardia Syndrome patients also have joint hypermobility. In these, a mechanism is hypothesized wherein collagen-related abnormalities in vascular elasticity and compliance can lead to peripheral vascular pooling which in turn exacerbates orthostatic intolerance related symptoms (Benarroch, 2012; Mathias et al., 2012).

**Table 3:** **Postural Tachycardia subtypes**.

**Mechanism of Postural Tachycardia Syndrome subtype**	**Markers**	**Examples**
Neuropathic Postural Tachycardia Syndrome: Impaired sympathetically mediated vasoconstriction in the lower limbs	Impaired distal sweating, blunted late phase ii in Valsalva maneuver, low supine blood pressure, reduced NA spillover in leg veins, reduced cardiac meta-iodobenzylguanidine uptake, High leg blood flow	Restricted postviral or autoimmune neuropathies
Hyperadrenergic Postural Tachycardia Syndrome: exaggerated cardiac sympathoexcitatory responses	Standing plasma NA ≥600 pg/mL, fluctuating blood pressure or hypertension during head up tilt	Anxiety, Pheochromocytoma, Mast cell activation disorders, voltage-gated potassium channel autoimmunity
Volume dysregulation	Elevated plasma angiotensin II, Impairment of renin-angiotensin-aldosterone system, impaired renal control of fluid secretion	Conditions associated with hypovolemia
Physical deconditioning	V_O2max_ ≤ 85% on exercise testing, reduced left ventricular mass	Prolonged bed rest, Chronic fatigue syndrome

*Overview of Postural Tachycardia Syndrome phenotypes, adapted from Benarroch (2012)*.

#### Association with psychiatric disorders

At the population level, very similar patterns are observed in the relationship between Postural Tachycardia Syndrome or Joint Hypermobility with anxiety disorders, particularly panic disorder (Bulbena et al., 2004c). For example in both, anxiety is significantly more common in young females (Martin-Santos et al., 1998). The combination of Postural Tachycardia Syndrome and syncope is very strongly associated with anxiety symptoms, (Linzer et al., 1991; Kapoor et al., 1995; Ventura et al., 2001; Kouakam et al., 2002). Moreover, reported day-to-day functional disability in Postural Tachycardia Syndrome patients is closely related to catastrophising thoughts, somatic hypervigilance and ultimately anxiety disorders and symptoms (Benrud-Larson et al., 2003; Raj, 2006; Masuki et al., 2007; Raj et al., 2009). Accompanying mood changes also enhance a phenomenological overlap with generalized anxiety disorder (Khurana, 2006; Raj, 2006; Mathias et al., 2012). Recent data suggests patients with POTS commonly present with symptoms of depression, elevated anxiety and increased anxiety sensitivity, particularly with regards to cardiac symptoms, and have a poorer health related quality of life in both the physical and mental health domains (Anderson et al., 2014).

Despite these data, one small American study of 21 Postural Tachycardia Syndrome patients and 18 controls failed to find increased prevalence of major depression or anxiety disorders in this small patient sample. However, the patients scored significantly higher on inattention and ADHD subscales than control subjects (Raj, 2006), arguable representing an impaired capacity to regulate emotions.

Although Postural Tachycardia Syndrome shares with anxiety and panic disorder many psychological (e.g., tremulousness, health anxiety, impaired concentration) and co-existing physiological (e.g., palpitations, tachycardia, chest pain, dyspnea) (Esler et al., 2004) symptomatology, a differentiating factor is that Postural Tachycardia Syndrome can be provoked by orthostatic challenges alone (Khurana and Setty, 1996; Khurana, 2006; Masuki et al., 2007).

Postural Tachycardia Syndrome patients' attentional and recall abilities are significantly poorer than controls, (see Anderson et al., 2014): Patients report symptoms that commonly occur in attention deficit hyperactivity disorder (ADHD), (Raj et al., 2009). Such associations may be due to shared abnormalities in the norepinephrine transporter. One feature is that inattention decreases with illness duration, likely due to adaptive or treatment responses. Importantly, as distinct from ADHD, hyperactive traits are absent in childhood.

Poor quality sleep, daytime sleepiness and fatigue have also been found to impair Postural Tachycardia Syndrome patients quality of life (Bagai et al., 2011). In Postural Tachycardia Syndrome patients with comorbid chronic fatigue syndrome, working memory, accuracy and information processing are impaired during orthostasis, yet the cause of this “brain fog” that is commonly reported in many Postural Tachycardia Syndrome patients, remains elusive, despite investigations into cerebral blood velocity, sleep quality or neurotransmitter function (Ocon, 2013; Ross et al., 2013). Recent evidence may provide further insight into this given the findings that patients with POTS performed worse in tests of current intellectual functioning (verbal and non-verbal IQ) and in measures of focused attention (digits forward) and short term memory (digits back). The test results were influenced largely by years of education and the underlying level of depression and anxiety (Anderson et al., 2014).

#### Evidence of brain-body interactions from neuroimaging

**Figure 3:**
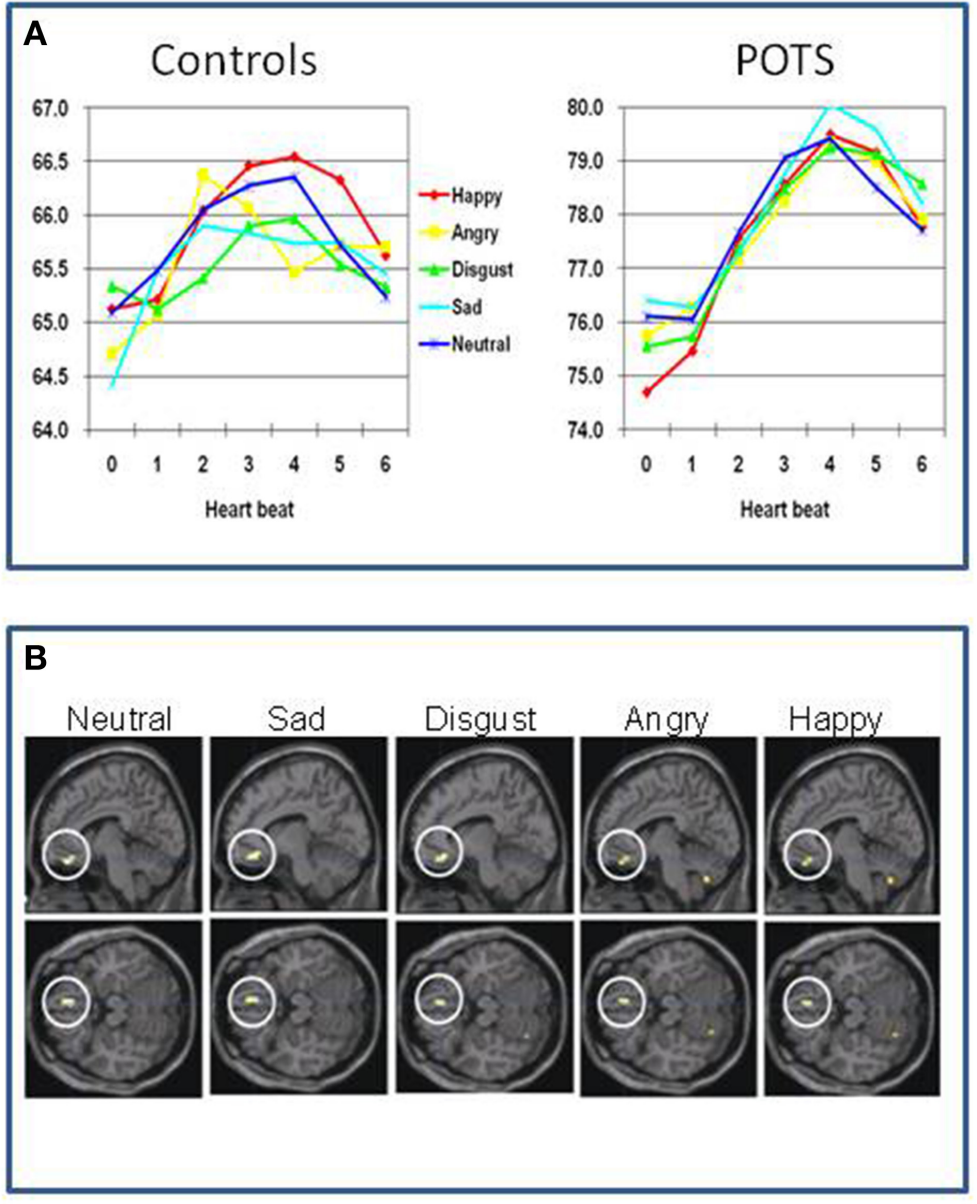
**Functional differences in brain of patients with postural orthostatic tachycardia syndrome (Postural Tachycardia Syndrome) (Umeda et al., 2009). (A)** Differences between controls and Postural Tachycardia Syndrome patients in resting heart rate and evoked responses to neutral and emotional stimuli, characterized by accentuated prolonged cardiac acceleration in orienting response with lack of emotion specificity. **(B)** Differences in regional brain activity between Postural Tachycardia Syndrome and control participants showing exaggerated withdrawal of activity within ventromedial prefrontal cortex in Postural Tachycardia Syndrome patients when encountering emotional or neutral stimuli.

Again, neuroimaging data in the field of Postural Tachycardia Syndrome is sparse. One study has examined twelve patients with Postural Tachycardia Syndrome and twelve matched controls (Umeda et al., 2009). Half the Postural Tachycardia Syndrome patients had joint hypermobility. Using functional imaging they examined the processing of emotional and neutral pictures. Physiologically, Postural Tachycardia Syndrome patients show exaggerated orientating responses compared to controls. Controls increase their heart rate when processing the visual stimuli by about 1.5 beats per minute peaking around 2–3 s after presentation. Postural Tachycardia Syndrome patients had a higher resting heart rate (76 compared to 65 beats per minute), yet showed an increase in heart rate to the pictures of 4–5 beats per minute, peaking around 4–5 s after stimulus onset. The orientating response in Postural Tachycardia Syndrome patients also did not show the same degree of sensitivity to the emotional content of the pictures observed in controls. This group difference was also reflected in differences in regional brain responses to the pictures (Figure 3). The deactivation of ventromedial prefrontal cortex, typically reflecting engagement during processing of external stimuli (and also implicated in “antisympathetic” autonomic control) was accentuated in Postural Tachycardia Syndrome patients irrespective of which emotion or neutral stimulus was presented. Also across both groups a region of the basal ganglia (globus pallidus) predicted state anxiety scores. In Postural Tachycardia Syndrome patients, connectivity between basal ganglia, orbital and dorsolateral prefrontal cortices reflected the interaction of anxiety state and physiological responsivity.

#### Conclusions

These findings endorse the proposal that Postural Tachycardia Syndrome patients are constitutionally vulnerable to anxiety symptoms through abnormalities in central mechanisms controlling autonomic reactions. Postural Tachycardia Syndrome patients showed generalized stimulus-evoked cardiovascular responses and did not show the distinct differences across emotion categories that was observed in controls (cardiac acceleration to anger and blunted cardiac response to disgust stimuli) and reported in previous studies (Ekman et al., 1983; Critchley, 2005). Thus, the exaggerated cardiovascular response observed in the Postural Tachycardia Syndrome patients abolishes the emotion-specificity of the psycho-physiological reactions. Overall these findings suggest that hyper-reactive bodily states can underlie the disruption of emotional state through attenuation of activity within VMPFC. The study of Umeda et al. (2009) improves understanding of why the expression of Postural Tachycardia Syndrome may increase vulnerability to panic anxiety and stress-sensitive psychosomatic disorders. These findings can help inform treatment strategies for the mitigation of mood and other symptoms in some Postural Tachycardia Syndrome, endorsing the view that approaches to correct sympathetic/parasympathetic balance (e.g., through exercise, Joyner and Masuki, 2008) or pharmacotherapy (e.g., beta-adrenoceptor blockade) to diminish the magnitude of cardio-accelerative responses (Esler et al., 2006) will impact on negative affective experience.

### Vasovagal syncope

#### Clinical descrpition and dysautonomia

Orthostasis should normally provoke an intricate integration of central and peripheral physiological feedback mechanisms to ensure maintained cerebral perfusion. Specifically, cerebral perfusion is dependent on arterial pressure, systemic vascular resistance, cardiac output, metabolic control and cerebrovascular resistance with intrinsic autoregulation. Unfortunately, neurovascular coupling allows for potential breakdown in this vital process. An identifiable clinical condition, such as autonomic failure, atrial fibrillation or ventricular arrhythmia, may cause syncope but when caused by vasodilatation and bradycardia, this type of syncope is defined as vasovagal syncope, the major form of autonomic mediated syncope (AMS).

Cerebral hypoperfusion resulting in transient loss of consciousness can be induced by a variety of physiological—or in some cases of vasovagal syncope, psychological–phenomena that lead to either inadequate cerebral perfusion pressure or abnormally high cerebral vascular resistance. Causes of syncope are heterogeneous but can be classified into three categories: Low arterial blood pressure; Increased resistance to cerebral blood flow; and other causes of cerebral dysfunction (see Table 4).

**Table 4:** **Classification and causes of syncope**.

**Low arterial blood pressure**	**Increased resistance to cerebral blood flow**	**Other causes of cerebral dysfunction**
Low cardiac output	Cerebral vasoconstriction	Epilepsy may be confused with simple faints
Inadequate venous return due to excessive venous pooling or low blood volume	Low Paco2, due to hyperventilation	Metabolic and endocrine disorders—hypoglycaemia, Addison's disease, hypopituitarism
Cardiac causes—tachyarrhythmias, bradyarrhythmias, valvular disease, bradycardia	Cerebral vasospasm	Electrolyte disorders—may be associated with hypovolaemia or predispose to cardiac arrhythmias
Low total peripheral vascular resistance	Vascular disease—either extracranial of intracranial areteries	
Vasovagal attacks		
Widespread cutaneous vasodilatation in thermal stress		
Reflex causes—vasovagal attacks, “carotid sinus syndrome,” visceral pain reflexes (may cause vasodilatation or vasoconstriction), decreased stimulation of visceral stretch receptors (e.g., voiding distended bladder)		
Vasodilator drugs		
Autonomic neuropathies		

The term “*vasovagal*” derives from the simultaneous vasodilatation and vagally-mediated bradycardia leading to hypotension and resultant transient loss in consciousness, and “*syncope*” descends from the Greek word, “*syncoptein*,” meaning to interrupt or cut short (Grubb and Karas, 1999). In 1932, Sir Thomas Lewis introduced the term “vasovagal attack”(Lewis, 1932) but “vasoregulatory asthenia” and “neurocirculatory asthenia” were first reported in soldiers during World War I due to poor neural regulation of peripheral blood flow perturbing cardiac function (Wooley, 1976). As Lewis described, vasovagal attacks are precipitated by an increase of sympathetic nerve activity– increased vascular resistance, blood pressure and heart rate—followed by bradycardia, vasodilation and a marked fall in arterial blood pressure, cerebral hypoperfusion.

Vasovagal syncope is the most common form of autonomic mediated syncope (other types include carotid and situational). It is characterized by a sudden transient loss of consciousness and postural tone caused by cerebral hypoperfusion provoked by physiological or emotional stressors. The term “vasovagal” derives from sympathetic vasoconstrictor withdrawal causing vasodilatation and increased vagal nerve activity causing bradycardia, these leading to hypotension and resultant loss of consciousness. vasovagal syncope accounts for around 40% of syncopal events, making it the most common cause of syncope (Fenton et al., 2000). It affects 15% of children under 18 years (Lewis and Dhala, 1999) while 35% of adults aged 35–60 experience a syncopal episode (Ganzeboom et al., 2006). Syncope accounts for 3–5% of emergency room admissions and evaluation of syncope cases accounts for 1–6% of hospital admissions (Kapoor et al., 1995), with a 35% chance of a recurrent episode (Savage et al., 1985). Fainting is typically triggered by social/emotional stress, often in the context of physical factors such as higher ambient temperature, prolonged standing, and body boundary violation (blood/injury) phobia. There may be a familial history of syncope (Mathias et al., 1998) and more than 80% of individuals will have their first episode before age 20 years. While benign, risks include injury from falling and hypoxic seizure due to hypotension.

#### Associations with psychiatric disorder

Psychiatry, neurology and cardiology research groups have found psychiatric conditions are over-represented in patients with vasovagal syncope, particularly anxiety, depression and somatization disorders (Giada et al., 2005). In psychiatry, a survey of 67 patients (Leftheriotis et al., 2008) identified 58% as showing a vasovagal episode during head up tilt and 45% had a history of syncope. Interestingly, vasovagal syncope patients who do not respond to treatment are more anxious on rat and depressed (on rating scales) than normal vasovagal syncope treatment responders and also report more negative thoughts regarding threats to physical harm or death as well as higher levels of avoidance/protection coping and rumination (Gracie et al., 2006).

Compared to non-fainting controls people with vasovagal syncope have higher rates of anxiety (30%) panic (20%), and depression (15%) and higher anxiety scores (Cohen et al., 2000; Kouakam et al., 2002). Recent work in women finds higher levels of anxiety in women with vasovagal syncope and controls with transient loss of consciousness compared to controls without (Zysko et al., 2014) Blood/injury phobia is common in vasovagal syncope (Luborsky et al., 1973; Sledge, 1978; Cohen et al., 2000; McGrady et al., 2001), with syncopal episodes often proceeding anticipation of real or fantasized physical harm in a social context, where fight/flight was perceived as unacceptable, i.e., “…*when an individual experiences fear he must deny”*(Engel, 1962). Depression, anxiety, frustration and helplessness are also identified antecedents of vasovagal syncope (Luborsky et al., 1973). Physical and psychosocial threat are a provocative factors for the induction of vasovagal syncope. Attenuated electrodermal stimulus processing negativity have been recorded during anticipation of unpleasant images in vasovagal syncope patients (Buodo et al., 2012), suggestive of a lack of emotional anticipation and adjustment to emotional stressors.

Faints are not only triggered by these salient emotional challenges but are also influenced by affective factors. However, the causal relationship between cognitive/affective style and vasovagal syncope is presently a subject of close scrutiny, and there exists a bidirectional relationship between cognitive/affective factors and syncopal symptoms in people with vasovagal syncope (Gracie et al., 2006).

Almost two thirds of patients referred for suspected syncope present with symptoms or clinical features of Postural Tachycardia Syndrome. Being female, under the age of 40 years, and with peripheral venous pooling when upright are among the diagnostic indicators. Autonomically mediated syncope and Postural Tachycardia Syndrome that are recorded previously (Stewart, 2009; Kanjwal et al., 2011) yet syncope occurs more commonly in Postural Tachycardia Syndrome (Ojha et al., 2010).

Further relationships exist between autonomic physiological parameters and the occurrence of fainting in people vulnerable to vasovagal syncope. In patients with vasovagal syncope, Low Frequency Heart Rate Variaibility is significantly negatively correlated with fainting frequency [*r* = −0.56, *p* = 0.018] and log [fainting frequency] [*F* = 12.64, *p* = 0.003, *R*2 = 0.46] in contrast, High Frequency Heart Rate Variability (predominately parasympathetic) was significantly positively correlated with fainting frequency [*r* = −0.54, *p* = 0.026] and log[fainting frequency] [*F* = 10.96, *p* = 0.005, *R*2 = 0.42] (Beacher et al., 2009).

#### Evidence of brain-body interactions from neuroimaging

**Figure 4:**
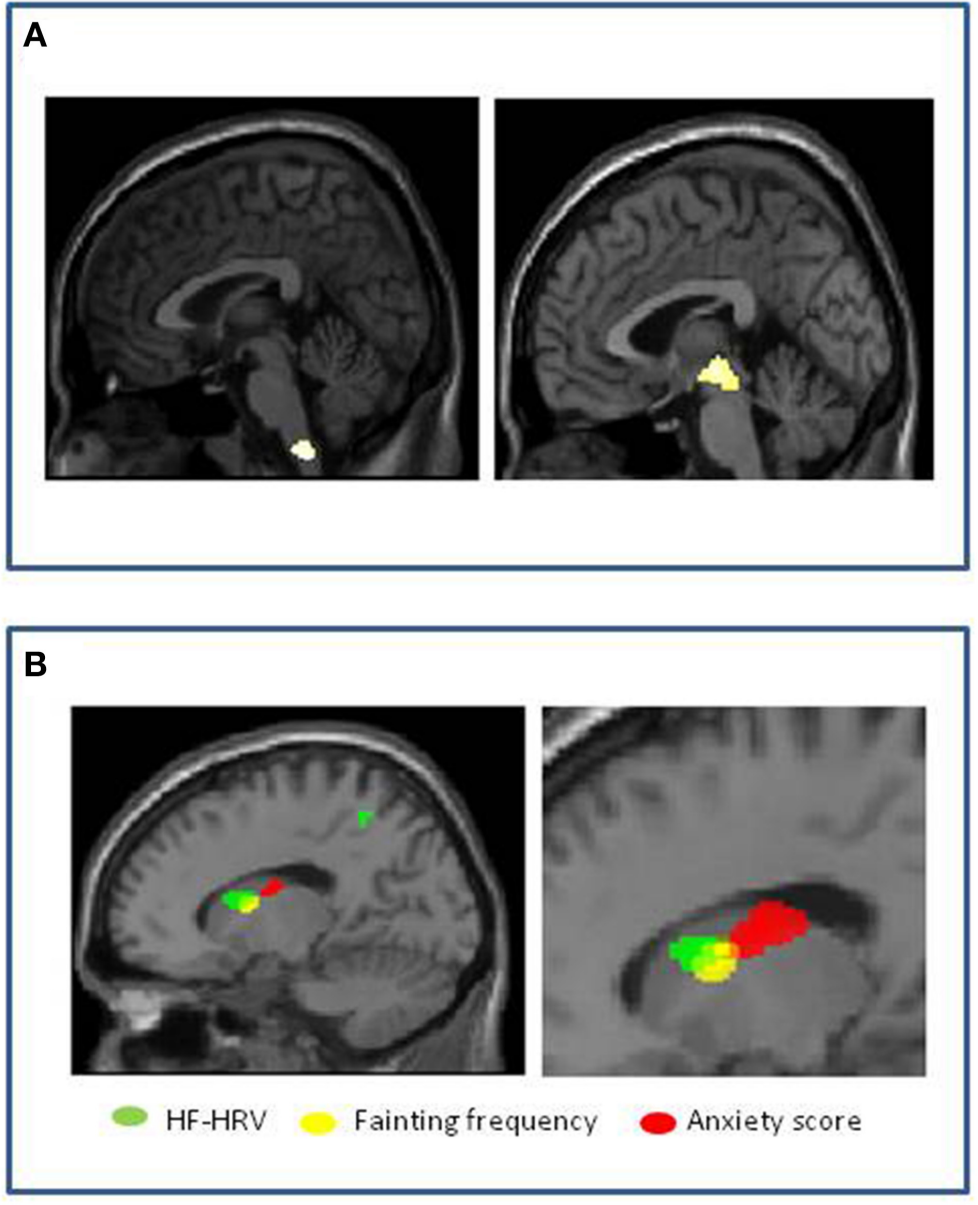
**Vasovagal syncope: Associations with brain structure (Beacher et al., 2009). (A)** Significant group differences in medulla and midbrain volumes between healthy people with and without history of fainting, with reduced volume associated with vasovagal syncope. **(B)** Correlation of volume within left caudate nucleus in people with history of fainting. Reduced gray matter volume in contiguous regions correlated with increased anxiety score (red), increased fainting frequency (yellow) and increased high frequency heart rate variability (HF-heart rateV) (green).

Using neuroimaging, Beacher et al tested for differences in brain structure between people that faint and those that do not, to understand more about the mechanisms governing underlying patterns of psychophysiological response and associated psychological vulnerabilities (Beacher et al., 2009). Using structural scans of healthy individuals with and without vasovagal syncope, they compared regional gray and white matter volumes across the whole brain, with special attention to brain stem regions (where neurodegeneration is associated with syncope in conditions like Mulltiple System Atrophy). They observed significant differences in regional volume (relative reduction in people with a history of fainting) within medulla and midbrain encompassing parts of the nucleus of the solitary tract (NTS) and ventral periaqueductal gray matter (vPAG) (Figure 4A). The bidirectional pathways between the PAG and the amygdala has been examined in animal studies of orienting and defense responses, with stimulation of the ventrolateral PAG (vlPAG) inducing passivity and down-regulation of sympathetic cardiovascular activity (Porges, 2003). Symbiotic autonomic changes, such as cardiovascular or sudomotor fluctuations accompany these behavioral modifications. Their findings were significant at the group level, but had less than 70% specificity and sensitivity; nevertheless they suggest that brainstem structure may be a context for vasovagal vulnerability. They also looked within the group as to the correlation with fainting frequency (this was in fact quite low across the group, a mean of 0.12 faints per year). Gray matter volume within the left caudate nucleus correlated with individual differences in lifetime number of fainting episodes. Interestingly, adjacent caudate regions correlated with anxiety scores and high frequency heart rate variability of these same individuals (Figure 4B).

#### Conclusions

Together these last observations suggest that interaction between emotional and physiological state at the level of caudate nucleus influence the likelihood of fainting predisposed to by differences in brainstem structural organization. These observations are noteworthy since an association is also demonstrated between high frequency or “vagal” regulation of the heart (HF-heart rateV) and frequency of syncope in vasovagal syncope participants. Moreover, emotional state, notably anxiety, is well recognized as a predisposing factor to syncope in vulnerable vasovagal syncope individuals (association was observed with the occurrence of fainting across all participants as a whole) and influences cardiovascular autonomic regulation (Friedman et al., 1993). Therefore, their interpretation of the findings is that predisposition to vasovagal syncope relates to differences in functional neuroarchitecture (expressed as differences in structural morphology) of brainstem regions mediating cardiovascular homeostasis, including baroreflex regulation of blood pressure. Further, their findings suggest that the likelihood of recurrent fainting is influenced at the level of left caudate nucleus, regions of which we found to be more strongly coupled to autonomic cardiac regulation in individuals with vasovagal syncope than in controls, and which interact with adjacent regions, also within the caudate, which contribute to the expression of anxiety.

### Additional phenotypes

#### Idiopathic hyperhidrosis

When the amount of excreted sweat exceeds that required to normalize body temperature and causes significant functional impairment, this is called “Idiopathic Hyperhidrosis.” The etiology of Idiopathic Hyperhidrosis remains unknown but the condition is defined by excessive local or generalized sweating, typically on the palms of the hands, soles of feet and axillary. Eccrine glands are primarily implicated in Idiopathic Hyperhidrosis, with the exception of axillary Idiopathic Hyperhidrosis which appears to have diffuse pathophysiology (Bovell et al., 2001; Lonsdale-Eccles et al., 2003). There can be a number of causes of secondary hyperhidrosis (see Table 1) and once these have been eliminated, sudomotor activity can be examined with indicator dyes that change color on exposure to moisture whilst the patient's body temperature is raised in a thermoregulatory sweat test (Low and Fealey, 2002). Idiopathic Hyperhidrosis can be provoked by everyday factors, such as mild exertion or food ingestion. Updated refs in 5th edition AF chapters.

Idiopathic hyperhidrosis patients commonly report anxiety (Karaca et al., 2007; Beacher et al., 2009), though it remains unclear whether anxiety is a prodromal symptom of IH or *vice versa* (Noppen et al., 1997). Explanations of Idiopathic Hyperhidrosis as basal sympathetic hyperactivity are complicated by most patients' accompanying affective distress and heart rate variability findings of increased parasympathetic heart rate variability (Birner et al., 2000; Kaya et al., 2005). In a study by De Marinis and co-workers (Kaya et al., 2005), 34% of Idiopathic hyperhidrosis patients also had orthostatic hypotension, furthermore, this sub-group had greater total body sweat rates and larger orthostatic blood pressure and heart rate changes than the remaining Idiopathic Hyperhidrosis patients and normal controls. However, in consideration of the subjects' age range (28 ± 6 years); these were more likely to be vasovagal episodes rather than autonomic failure-induced hypotensive episodes.

Surgical interventions for Idiopathic Hyperhidrosis typically involve thoracic sympathectomy, which often causes compensatory sweating yet still apparently improves psychosocial distress (Ramos et al., 2006). Such convoluted findings make delineating emotional and sudomotor factors in Idiopathic Hyperhidrosis challenging.

#### Depersonalization disorder

Depersonalization disorder is a dissociative disorder, defined by symptoms that include feelings of derealization (one's surroundings feel unreal), emotional numbing, feelings of disembodiment and memory recall deficits relating to the personalization but not retrieval of memory (Lee et al., 2012). Depersonalization is a disengaging defense response to overwhelming threat (Lee et al., 2012), with a lifetime prevalence of 74% for mild episodes and 1–2% for chronic Depersonalization disorder (Sierra and David, 2011).

Despite significantly elevated levels of anxiety in depersonalization disorder (Sierra et al., 2012), subjects have consistently recorded attenuated sympathetic responses to emotional aversion. Lader and Wing (1966) recorded the results of two panic disorder patients who became depersonalized during skin conductance recording. In their anxious or panicked state, the patients displayed low skin resistance but during episodes of depersonalization, both recorded a lack of skin conductance fluctuations and significant increases in skin resistance. Compared to “agitated depressed, non-agitated depressed, schizophrenic, phobic, obsessive compulsive disorder, personality disorder, hysteria” patients and normal controls, depersonalization disorder patients had the lowest baseline measure of forearm blood flow of all the groups (Kelly and Walter, 1968).

Electrodermal responses have predominantly been used as a peripheral measure of autonomic arousal in depersonalization Simultaneous recording of electrodermal responses (sympathetic drive to skin) with functional neuroimaging, finds that depersonalization disorder patients produce decreased hypothalamic and amygdala blood oxygen level dependent (BOLD) responses during presentation of happy and sad expressions respectively (Lemche et al., 2007, 2008). These findings were reversed in healthy controls. Electrodermal responses are both more quickly manifested and yet abnormally weakened in Depersonalization disorder participants during aversive stimuli exposure (Sierra et al., 2002), indicating hypervigilant attentional appraisal and rapid suppression of physiological emotional arousal. Norepinephrine levels have also been found to be negatively correlated with Depersonalization disorder severity (Simeon et al., 2003).

Depersonalization disorder patients share anecdotal symptoms with patients with corticolimbic disconnections (Mayer-Gross, 1935), supporting the hypothesis that, in Depersonalization disorder, emotional manifestation and allocation has become out-of-step with the processes required for emotional embodiment. Models of Depersonalization disorder, posit environmental and emotional hypervigilance and subsequent emotional attenuation during emotional aversion, evidenced by reduced electrodermal responses to disagreeable images compared to both healthy controls and anxiety disorder patients, despite the Depersonalization disorder subjects being equally as anxious as the anxiety participants (Sierra et al., 2002).

In Depersonalization disorder, inverse correlations between right ventral PFC BOLD activity and left insular BOLD responses during aversive emotional stimuli have been reported (Phillips et al., 2001), and between happy and sad expression intensity and right hypothalamus and right amygdala BOLD responses respectively have been recorded. Moreover, negative correlations between SCRs and dorsal PFC BOLD responses have also been described in Depersonalization disorder (Lemche et al., 2007, 2008).

## Conclusions

Together these data argue for the recognition of neurovisceral phenotypes that underlie constitutional predisposition to affective symptoms and that reflect the interaction and emergence of affective feelings with representations and control of bodily arousal. There is circumstantial evidence for a genetic basis to some neurovisceral phenotypes, such as in relation to certain collagen disorders. Neuroimaging techniques, particularly when combined with physiological measurement, have the potential to dissect brain mechanisms mediating the interplay between physiological control and psychological response to emotion. Data in neurovisceral phenotype models implicate a discrete set of brain regions that include the amygdala, cingulate and insula cortex along with specific levels of the brainstem, basal ganglia and ventromedial prefrontal cortex. Further work is needed to extend what is already known regarding the distinct and overlapping contribution of these regions to subjective feelings of anxiety, perception and misperception of bodily arousal, generation of stereotyped patterns of affective reactions (faints, tachycardia, sweating and hyperventilation). A detailed understanding of these processes in clinical and non-clinical groups, alongside greater clinical recognition of neurovisceral phenotypes and their expression across neuropsychiatric disorders, will inform the innovative development of pharmacological and biobehavioral interventions for the management of psychosomatic symptoms, panic and anxiety states.

### Conflict of interest statement

The authors declare that the research was conducted in the absence of any commercial or financial relationships that could be construed as a potential conflict of interest.
